# Factors Associated with Skin Cancers in People with Albinism in Togo

**DOI:** 10.1155/2021/3433493

**Published:** 2021-12-23

**Authors:** Abas Mouhari-Toure, Sefako Abla Akakpo, Julienne Noude Teclessou, Piham Gnossike, Saliou Adam, Garba Mahamadou, Panawé Kassang, Yvette Elegbede, Tchin Darre, Koussake Kombate, Palokinam Pitché, Bayaki Saka

**Affiliations:** ^1^Service de Dermatolgie et IST, CHU Kara, Université de Kara, Kara, Togo; ^2^Service de Dermatolgie et IST, CHU Sylvanus Olympio, Université de Lomé, Lomé, Togo; ^3^Service de Dermatolgie et IST, CHU Campus Université de Lomé, Lomé, Togo; ^4^Centre National de Dermatologie de Gbossimé, Lomé, Togo; ^5^Service de Chirurgie Maxillo-faciale et Plastique, CHU Sylvanus Olympio, Université de Lomé, Lomé, Togo; ^6^Service de Dermatolgie et IST, Centre Hospitalier Régional de Tomdè, Kara, Togo; ^7^Laboratoire D'anatomie et Cytotologie Pathologique, CHU Sylvanus Olympio, Université de Lomé, Lomé, Togo

## Abstract

**Objective:**

The aim of this study was to identify the factors associated with skin cancers in people with albinism (PWA) in Togo.

**Method:**

This is a retrospective analytical study of the records of PWA examined during five dermatological consultation campaigns from 2019 to 2021.

**Results:**

During the study period, 517 PWA were seen. Sixty-four (12.3%) of these PWA had presented with 137 cases of skin cancer. The sex ratio (M/F) was 0.9. The average age of PWA with skin cancer was 39.69 ± 15.61 years and that of PWA without skin cancer was 19.17 ± 15.24 years (*p* ≤ 0.001). The 137 cases of skin cancers were dominated by basal cell carcinomas (45.9%). These skin cancers were located preferentially in the cephalic region (77 cases; 56.2%), followed by the upper limbs (33 cases; 24.1%). In multivariate analysis, the risk factors for skin cancers in PWA were age over 39 years (*p* ≤ 0.001) and the presence of actinic keratoses (*p* ≤ 0.001). In contrast, the presence of ephelides (*p*=0.018) was a protective factor.

**Conclusion:**

This study confirms that advanced age and actinic keratoses are risk factors for skin cancer in PWA, in connection with the cumulative role of solar radiation. Its originality lies in the identification of ephelides as a protective factor. The knowledge and consideration of these risk factors will make it possible to optimise strategies for the prevention of skin cancers in PWA.

## 1. Introduction

Oculocutaneous albinism (OCA) is an inherited condition caused by a deficiency or absence of tyrosinase activity, a key enzyme in melanin biosynthesis by melanocytes in the skin, hair follicles, and eyes [1, 2]. Because of the reduction or absence of melanin, PWA are very sensitive to the harmful effects of ultraviolet (UV) light. They are more at risk of actinic lesions and dermatoses, the most worrying of which are precancerous and cancerous lesions [3]. Epidemiologically, OCA is a universal and ubiquitous condition affecting approximately 1/17,000 people worldwide regardless of race or gender [4]. In sub-Saharan Africa, it is about 1/5000 people [5]. African PWA are more prone to skin cancers because they live near the equator where exposure to the Sun's UV rays is very high [6]. According to the United Nations, 98% of PWA do not live beyond the age of 40 because of sun exposure, with skin cancers accounting for at least 80% of their deaths [5]. The majority of these skin cancers are represented by squamous cell carcinoma (SCC) in 75–88%, basal cell carcinoma (BCC) in 9–23%, and melanoma in rare cases (1.3–3%) [7]. Genetic (phototypes) and environmental (sun exposure) factors have been implicated in the occurrence of non-melanoma skin cancers [8, 9].

Some studies have reportedly shown a correlation between the presence of ephelides and the age of PWA [10, 11]. Studies conducted in Africa on PWA are mostly descriptive, as in Togo [12, 13]. We are conducting this study to identify the risk factors associated with skin cancers in PWA in Togo, in order to propose adequate preventive measures.

## 2. Method

This is a retrospective analytical study of the files of PWA examined during five dermatological consultation campaigns from 2019 to 2021, as part of a programme for the prevention and management of skin cancers in these subjects. For each campaign, there was a coordination team from the National Association of People with Albinism in Togo (ANAT) and a medical team made up of dermatologists proposed by the Togolese Society of Dermatology and Sexually Transmitted Infections (SOTODERM), a maxillofacial surgeon, and the ANAT health manager. The number of dermatologists during these campaigns varied depending on whether one was in the capital Lomé (2 to 3 dermatologists for three days) or in a town in the interior (one dermatologist for 2 days in Atakpamé and one dermatologist for one day in the rest of the towns visited). The diagnosis of albinism was exclusively clinical, based on the colour of the skin and hair (generalized hypopigmentation of the skin and hair) and the presence of nystagmus. During these campaigns, when a lesion was suspected of being cancerous, a biopsy or biopsy-exeresis was performed and sent to the pathological anatomy laboratory of the Mélia clinic or the CHU Sylvanus Olympio. For each PWA, the data collected were sociodemographic (age, sex, and place of residence), clinical, and histological (precancerous lesions, existence and type of skin cancers, localization, and histology results).

### 2.1. Data Processing and Statistical Analysis

The questionnaires were entered using CS software version 7.6. The entered data were then exported to a database in Excel format. Descriptive analyses were carried out, and the results were presented in the form of graphs, tables, frequencies, and percentages. Quantitative variables were described by means (±standard deviation), and qualitative variables were described by frequencies and percentages. A bivariate analysis was performed. Quantitative variables were compared by Student's *t*-test, and qualitative variables were compared by the chi^2^ test. Variables that were associated with skin cancer risk factors (probability values less than 0.25) were retained for multivariate analysis. The multivariate analysis was therefore performed for variables significantly associated with skin cancers. The results expressed as odds ratio (OR) were given with a 95% confidence interval. All analyses were performed using R© version 3.3.2 software.

## 3. Results

During the 5 campaigns, 517 PWA were consulted at least once, of which 64 (12.4%) had presented with 137 cases of skin cancer (the number of skin cancers varied from 1 to 10 in one patient). The mean age of the PWA was 21.7 ± 16.7 years (range: 1 and 90 years) with a sex ratio (M/F) of 0.9. PWA with skin cancer had a mean age of 39.7 ± 15.6 years, and PWA without skin cancer had a mean age of 19.2 ± 15.2 years (*p* ≤ 0.001). A family history of albinism was found in 196 PWA (37.9%), and consanguinity was reported in 85 PWA (16.4%). The PWA were more likely to live in urban areas (78.9%).

The majority of PWA (*n* = 399; 77.2%) had freckles (ephelides), followed by actinic keratoses (*n* = 298; 57.6%), actinic cheilitis (*n* = 239; 46.2%), and elastosis (*n* = 4; 0.8%). The 137 cases of skin cancer were dominated by BCC (*n* = 63; 45.9%) (a patient could have several cases and histological types of skin cancer) (Table 1). The skin cancers were located in the cephalic region (*n* = 77; 56.2%), followed by the upper limbs (*n* = 33; 24.1%), trunk (*n* = 21; 15.3%), and lower limbs (*n* = 6; 4.4%). In univariate analysis, age above 39 years, the existence of ephelides, and actinic keratoses were associated with the risk of skin cancer (Table 2). In multivariate analysis, the risk factors for skin cancers in PWA were age over 39 years (*p* ≤ 0.001) and the presence of actinic keratoses (*p* ≤ 0.001). In contrast, the presence of ephelides (*p*=0.018) was a protective factor (Table 3).

## 4. Discussion

In our study, the prevalence of skin cancer was 12.4%. To our knowledge, no work has documented the incidence or the prevalence of skin cancers in the non-albino population in Togo for comparison with the albino one. This prevalence (12.4%) is lower than the 26% reported in Brazil [14], 25% in Tanzania [15], 23% in South Africa [10], and 20.9% in Nigeria [16] but higher than 4.6% reported in France [17]. Our low rate can be explained by the absence of some PWA during the campaigns because they live in very remote areas that have not been affected by these activities and have no access to health centres. The mobile consultation strategy is certainly the one we used during the campaigns, but our consultations mainly reach PWA in urban areas. Furthermore, PWA are the target of prejudice and social exclusion, which would prevent them from having easy access to health care and information about mobile consultations [14, 18].

The average age of onset of skin cancer in PWA in our series was 39.5 years, lower than that of the general Togolese population (42 years) [19]. PWA develop solar radiation-related diseases earlier because of the lack of sun protection from a young age, and cancerous tumours occur from the second decade of their lives [4, 20]. This cumulative role of solar radiation also explains the fact that the average age of PWA with skin cancer was significantly higher than that of PWA without skin cancer in our study. Thus, photoprotection remains a challenge from the young age of PWA, especially as the dress code is light due to the hot climate.

We counted 137 cases of skin cancers dominated by basal cell carcinomas. The predominance of BCC could therefore be explained by the fact that our screening was active during the campaigns, with biopsy of all suspected lesions, even asymptomatic ones. In Nigeria [16], BCC was also the most frequent skin carcinoma (55%), followed by SCC (22%). In addition to cutaneous carcinoma, we found cases of melanoma, lymphoma, and Paget's disease, which we did not find in 2019 [13], probably due to the limited number of patients.

In our study, the risk factors for skin cancer in PWA were age above 39 years (*p* ≤ 0.001) and the presence of actinic keratoses (*p* ≤ 0.001), which is related to the cumulative role of solar radiation. In contrast, the presence of ephelides (*p*=0.018) was a protective factor.

Regarding age, PWA aged 39 years and over were the most represented among those with skin cancers. Indeed, the risk of developing skin cancer in PWA was multiplied by 6.32 as age increased. This finding was also made in the Brazilian series which also highlighted the influence of age in the occurrence of skin cancer in PWA [14]. The main reason would be the effect of early skin ageing due to cumulative exposure to solar radiation. For technical reasons (unavailability of photobiology), we could not quantify this UV exposure in our PWA.

Secondly, the presence of actinic keratoses multiplies the risk of developing skin cancer in PWA by 4.639. The presence of actinic keratoses has already been found to be a risk factor associated with the occurrence of skin cancers in the Brazilian study [14]. Actinic keratoses are in fact precancerous lesions, and some of them are even carcinomas in situ.

Furthermore, the presence of ephelides reduces the risk of developing skin cancer by more than 1.63 times. This finding of a protective factor for ephelides was made by Kromberg et al. [10] but is difficult to interpret.

## 5. Limitations of the Study

The main strength of our study is that it is the doctors who travel to the populations, which solves some of the problems that are limitations of hospital-based studies, notably geographical inaccessibility and lack of financial means to consult. Its main limitation was its retrospective nature and the sincerity of the information collected from the PWA during the campaigns.

## 6. Conclusion

This study confirms that advanced age and actinic keratoses are risk factors for skin cancer in PWA, in connection with the cumulative role of solar radiation. Its originality lies in the identification of ephelides as a protective factor. The knowledge and consideration of these risk factors will make it possible to optimise strategies for the prevention of skin cancers in PWA.

## Figures and Tables

**Table 1:** Frequency of the main skin cancers.

	Number	%
Basal cell carcinoma	63	45.9
Squamous cell carcinoma	55	40.1
Bowen disease	12	8.8
Malignant melanoma	2	1.5
Paget's disease	2	1.5
Malignant lymphoma	3	2.2
Total	137	100

**Table 2:** Factors associated with skin cancer in univariate analysis.

	PWA without skin cancer	PWA with skin cancer	Total	*p* value
	*N* = 453	*N* = 64	*N* = 517	
Age (years)				
Mean (standard deviation)	19.2 (±15.2)	39.7 (±15.7)		≤0.001
>39, *n* (%)	45 (58.4)	32 (41.6)	77	≤0.001
≤39, *n* (%)	408 (92.7)	32 (7.3)	440	
Sex				0.989
Female, *n* (%)	234 (87.6)	33 (12.4)	267	
Male, *n* (%)	219 (87.6)	31 (12.4)	250	
Area of residence				0.071
Rural, *n* (%)	90 (82.6)	19 (17.4)	109	
Urban, *n* (%)	363 (89.0)	45 (11.0)	408	
Presence of ephelides				0.042
No	97 (82.2)	21 (17.8)	118	
Yes	356 (89.2)	43 (10.8)	399	
Presence of elastosis				0.450
No	449 (87.5)	64 (12.5)	513	
Yes	4 (100.0)	0 (0.0)	4	
Presence actinic keratosis				≤0.001
No	210 (95.9)	9 (4.1)	219	
Yes	243 (81.5)	55 (18.5)	298	

**Table 3:** Risk factors associated with skin cancer in PWA (multivariate analysis).

Variables	aOR	IC95%	*p* value
Age (years)			
>39	1.000	—	—
≤39	0.163	0.09–0.3	≤0.001
Area of residence			
Urban	1.000	—	—
Rural	1.598	0.84–3.04	0.063
Presence of ephelides			
No	1.000	—	—
Yes	0.462	0.24–0.88	0.018
Presence of actinic keratosis			
No	1.000	—	—
Yes	4.321	2.11–8.87	≤0.001

## Data Availability

The data used to support the findings of this study are available from the corresponding author upon request.

## Abbreviations

ANAT: National Association of Albinos of Togo
BCC: Basal cell carcinoma
PWA: Patients with albinism
SCC: Squamous cell carcinoma
SOTODERM: Togolese Society of Dermatology and Sexually Transmitted Infections
UV: Ultraviolet.
